# Supplementary material for: Evaluating the Performance of a Magnetic Nanoparticle-Based Detection Method Using Circle-to-Circle Amplification
Source: Biosensors (Basel). 2021 May 28;11(6):173. doi: 10.3390/bios11060173 (PMC8226732; doi:10.3390/bios11060173)
Supplement: Supplementary file 1 [file biosensors-11-00173-s001.zip › biosensors-1217230-supplementary.pdf]

Evaluating the performance of a magnetic nanoparticle-based detection method using circle-to-circle amplification

Darío Sánchez Martín[1,*], Reinier Oropesa-Nuñez[2,*], Teresa Zardán Gómez de la Torre[1]

[1] Department of Material Sciences and Engineering, Division of Nanotechnology and Functional Materials, Uppsala University, Ångström Laboratory, Box 534, SE-751 21 Uppsala, Sweden

[2] Department of Material Sciences and Engineering, Division of Solid-State Physics, Uppsala University, Ångström Laboratory, Box 534, SE-751 21 Uppsala, Sweden

[*]These authors contributed equally to this work.

Table S1. Sequences used in this study.

	**Sequence (5′-3′)**
**Padlock probe NDV**	Phos-TGACCCCATTTCCCTGGTGTATGCAGCTCCTCAGTAATAGTGTCTTACGACGCAACTTCACCGAATGAAAGGATATGCTATCYTGAA
**Target NDV**	Biotin-CTCTCTCTCTCAGGGAAATGGGGTCATTCARGATAGCATATCCTT
**Restriction oligo NDV**	GTGTATGCAGCTCCTCAGTA
**Detection oligo NDV for 1RCA**	Biotin-TTTTTCCTCAGTAATAGTGTCTTAC-6-FAM
**Detection oligo NDV for C2CA**	Biotin-TTTTTCCTCAGTAATAGTGTCTTAC
**Padlock probe *Salmonella***	Phos-GAACATAGAAATGATCATCGTGTATGCAGCTCCTCAGTAATAGTGTCTTACCAGTCGCGTGAGGACCAAATAGGTAGGTAATGGRATGAC
**Target *Salmonella***	GATGATCATTTCTATGTTCGTCATYCCATTACCTACCT
**Capture oligo *Salmonella***	Biotin-CTCTCTCTCTCTCTCTCTCTGTAATTCAGGTCGTAAACGAGCACTGTTAAGTAGAGAAAG
**Restriction oligo *Salmonella***	GTGTATGCAGCTCCTCAGTA
**Detection oligo *Salmonella* for C2CA**	Biotin-TTTTTGTAAGACACTATTACTGAGG

Magnetization spectra for 1RCA NDV samples

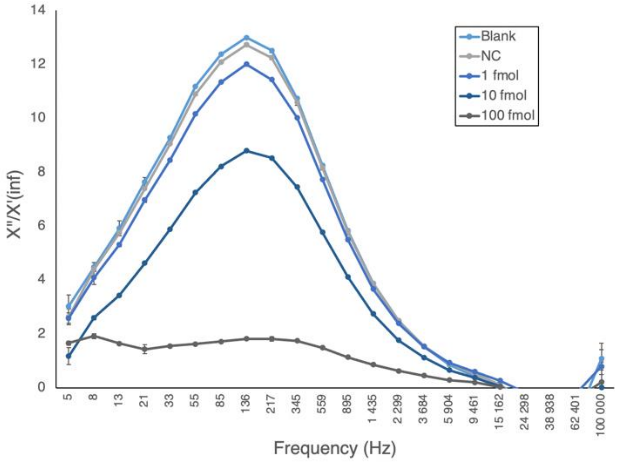

Figure S1. χ''/ χ'$_{inf}$ part of the magnetization spectra of the experiments presented in Figure 2. Error bars represent standard deviation based on two independent samples. The peak values for the different samples are the following: Blank = 12.99±0.02, NC = 12.72±0.05, 1 fmol = 12.00±0.01, 10 fmol = 8.79±0.0035 and 100 fmol = 1.85±0.03.

Magnetization spectra for C2CA NDV samples

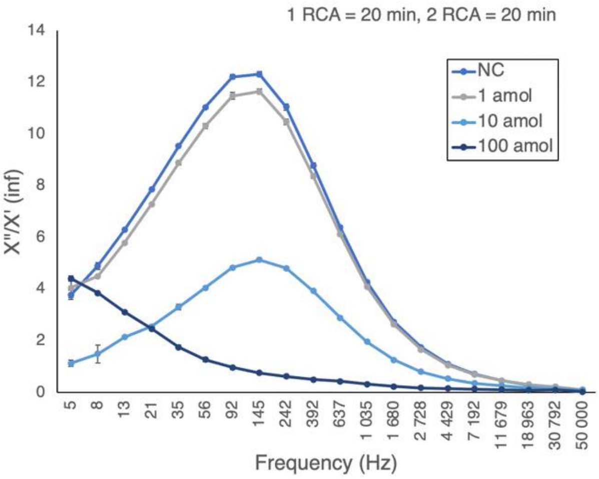

Figure S2. χ''/ χ'$_{inf}$ part of the magnetization spectra of the experiments presented in Figure 3 with the following amplification times: 1RCA = 20 min and 2RCA = 20 min. Error bars represent standard deviation based on two independent samples. The peak values for the different samples are the following: NC = 12.31±0.1, 1 amol = 11.64±0.11, 10 amol = 5.13±0.07 and 100 amol = 0.76±0.03.

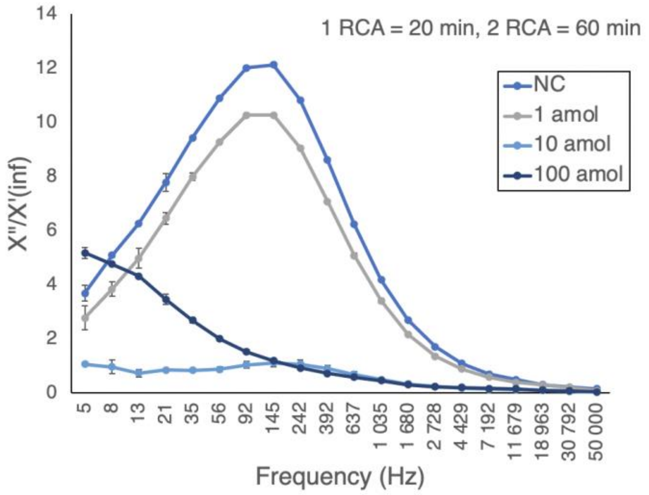

Figure S3. χ''/χ'_{inf} part of the magnetization spectra of the experiments presented in Figure 4 with the following amplification times: 1RCA = 20 min and 2RCA = 60 min. Error bars represent standard deviation based on two independent samples. The peak values for the different samples are the following: NC = 12.12±0.08, 1 amol = 10.26±0.02, 10 amol = 1.1±0.15 and 100 amol = 1.19±0.05.

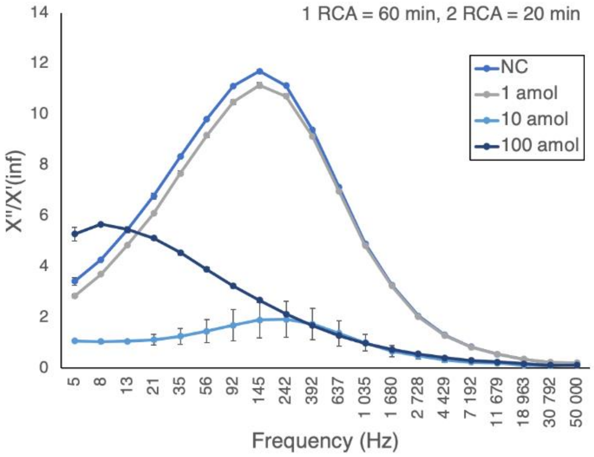

Figure S4. χ''/χ'_{inf} part of the magnetization spectra of the experiments presented in Figure 4 with the following amplification times: 1RCA = 60 min and 2RCA = 20 min. Error bars represent standard deviation based on two independent samples. The peak values for the different samples are the following: NC = 11.70±0.05, 1 amol = 11.14±0.10, 10 amol = 1.9±0.69 and 100 amol = 2.68±0.07.

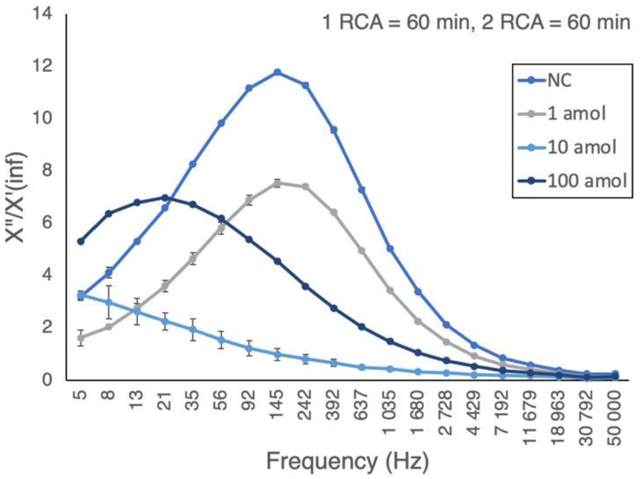

Figure S5. χ''/χ'_{inf} part of the magnetization spectra of the experiments presented in Figure 4 with the following amplification times: 1RCA = 60 min and 2RCA = 60 min. Error bars represent standard deviation based on two independent samples. The peak values for the different samples are the following: NC = 10.08±0.25, 1 amol = 7.53±0.15, 10 amol = 0.99±0.22 and 100 amol = 4.56±0.01.

Magnetization spectra for C2CA *Salmonella* samples

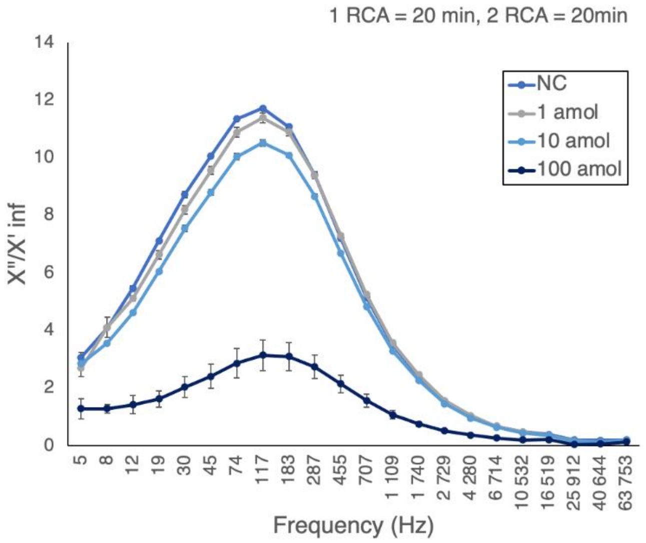

Figure S6. χ''/χ'_{inf} part of the magnetization spectra of the experiments presented in Figure 5 with the following amplification times: 1RCA = 20 min and 2RCA = 20 min. Error bars represent standard deviation based on two independent samples. The peak values for the different samples are the following: NC = 11.72±0.018, 1 amol = 11.38±0.16, 10 amol = 10.51±0.11 and 100 amol = 3.14±0.52.

Magnetization spectra for C2CA biological samples spiked with synthetic NDV target

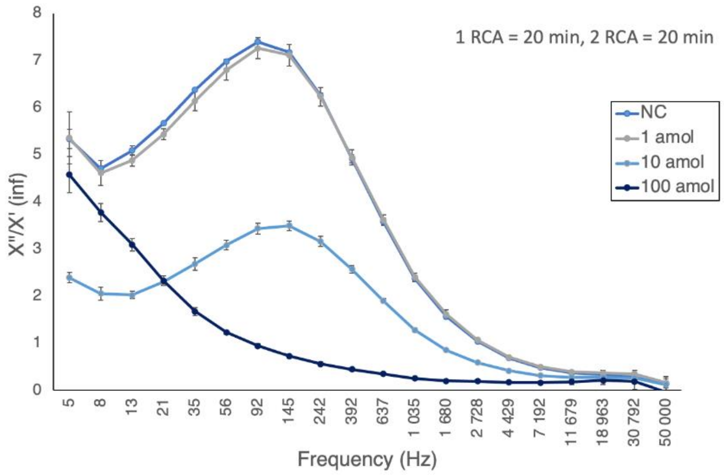

Figure S7. χ''/χ'_{inf} part of the magnetization spectra of the experiments presented in Figure 6 with the following amplification times: 1RCA = 20 min and 2RCA = 20 min. Error bars represent standard deviation based on three independent samples. The peak values for the different samples are the following: NC = 7.39±0.04, 1 amol = 7.25±0.22, 10 amol = 3.43±0.10 and 100 amol = 0.96±0.04.
